# London Homœopathic Hospital

**Published:** 1902-05-17

**Authors:** 


### Around the Hospitals.

### LONDON HOMŒOPATHIC HOSPITAL.

The annual report of this hospital shows that the in-patients numbered 1,092, and the out-patients (including 13,144 renewals), 21,822. The out-patients' attendances numbered 39,871. The accounts show that the ordinary income totalled £7,278, and the extraordinary £3,684. Legacies:—Mr. E. Roche, £500; Mrs. E. A. Wallis, £184. Endowed beds:—Margaret Tyler bed, £1,000; Helen Kingsbury bed, £1,000; James Cundy bed, £1,000. The ordinary expenditure was £10,957, and an extraordinary amount of £133 was spent on repairs and electric-light fittings. The deficit on ordinary income was thus £3,679, making, with £5,995 brought forward, a total due to capital of £9,674. This expenditure of capital funds received, says the report, has the special sanction of the meeting in 1900, which empowered the board to withdraw from the reserve fund the sum of £3,000 per year for the four years ending December 31st, 1902. The income in 1901, though £1,524 less than in 1900, is greater than in any previous year, some specially large donations having been received in 1900. The receipts from nursing fees—£923—have been less than in any previous year since 1897, but would have been greater had it not been for the fact that five fully-trained nurses, chosen for the Army Nursing Service, are still on duty in South Africa. The board points out that such munificent help as arose from the generosity of a few donors in 1900 could hardly be looked for from year to year, but they feel that the marked increase in the average of income is calculated to inspire hope as to the future. Nevertheless, the decrease, however temporary, of income in 1901, in conjunction with an increase of expenditure, has caused a considerable deficit, and as the expenditure cannot be reduced without lessening efficiency or hampering the activities of the medical staff, the board again appeals earnestly for increased support. During the year a new departure has been made in the organisation of "The Ladies' Guild" of helpers of the hospital. Inaugurated by a drawing-room meeting at the invitation of the Countess Cawdor, it has been followed up by the formation of a very vigorous centre at Hampstead, under the presidency of Mrs. Fellowes Pearson, and the management, as honorary secretary, of Mrs. E. W. Kimber. Other centres are in process of formation, and it is anticipated that "The Ladies' Guild of the Hospital" will render important aid both in respect of annual subscriptions and also by providing gifts of clothing for the patients in the wards. The board records with great regret the death of Mr. Jas. Slater, for many years a member of the board, and the death of Lady Newton, for many years a visitor to the wards. The hospital has also lost by death during the year the lady who, under the name of "A friend well known to the Hospital," as well as under her own name, has for many years been the donor of the largest gifts which the hospital has ever received from a living donor. Miss J. Durning Smith, widely known and esteemed not only for her constant generosity, but for her many gracious qualities, contributed to the hospital funds during the last twenty years a total sum of not less than twenty thousand pounds. In addition she was a regular visitor to the patients in the wards, and her loss is one which is very severely felt. The managers have commemorated her interest in the hospital by a tablet in the ward named by them at her request the Durning ward, and at their request, Sir Edward and Lady Durning-Lawrence have presented the hospital a medallion in marble by the late Mr. Adams-Acton and a portrait of the deceased lady. As to institutional changes, Dr. Lestock Reid has resigned his post of assistant physician. The board has appointed Dr. Grantham Hill and Dr. James Searson, assistant physicians, and Mr. Austin E. Reynolds, assistant surgeon for diseases of the eye. Mr. J. O. Butcher has resigned his post of surgeon dentist, and the board has discontinued that department for the present. The electrical department has been developed under Dr. J. T. Ashton so as to include the treatment of lupus and other diseases. In the out-patient department a section has been opened on the strong recommendation of the medical staff for Ling exercises, and it is hoped that much good will result in cases of malformation or defective development in children. The annual report of the Homœopathic Convalescent Home at Eastbourne shows a total of 167 residents during 1901.

